# AI-Driven Image Analysis in Central Nervous System Tumors-Traditional Machine Learning, Deep Learning and Hybrid Models

**DOI:** 10.26502/jbb.2642-91280046

**Published:** 2022-01-10

**Authors:** AV Krauze, Y Zhuge, R Zhao, E Tasci, K Camphausen

**Affiliations:** 1 Center for Cancer Research, National Cancer Institute, NIH, Building 10, Room B2-3637, Bethesda, USA; 2 University of British Columbia, Faculty of Medicine, 317 - 2194 Health Sciences Mall, Vancouver, Canada

## Abstract

The interpretation of imaging in medicine in general and in oncology specifically remains problematic due to several limitations which include the need to incorporate detailed clinical history, patient and disease-specific history, clinical exam features, previous and ongoing treatment, and account for the dependency on reproducible human interpretation of multiple factors with incomplete data linkage. To standardize reporting, minimize bias, expedite management, and improve outcomes, the use of Artificial Intelligence (AI) has gained significant prominence in imaging analysis. In oncology, AI methods have as a result been explored in most cancer types with ongoing progress in employing AI towards imaging for oncology treatment, assessing treatment response, and understanding and communicating prognosis. Challenges remain with limited available data sets, variability in imaging changes over time augmented by a growing heterogeneity in analysis approaches. We review the imaging analysis workflow and examine how hand-crafted features also referred to as traditional Machine Learning (ML), Deep Learning (DL) approaches, and hybrid analyses, are being employed in AI-driven imaging analysis in central nervous system tumors. ML, DL, and hybrid approaches coexist, and their combination may produce superior results although data in this space is as yet novel, and conclusions and pitfalls have yet to be fully explored. We note the growing technical complexities that may become increasingly separated from the clinic and enforce the acute need for clinician engagement to guide progress and ensure that conclusions derived from AI-driven imaging analysis reflect that same level of scrutiny lent to other avenues of clinical research.

## 1. Introduction

The interpretation of imaging in medicine in general and in oncology specifically remains problematic due to several limitations which include the need for detailed clinical history, patient and disease-specific history, clinical exam features, previous and ongoing treatment, and the dependency on reproducible human interpretation when multiple factors are involved, and data sources are incompletely linked. To standardize reporting, minimize human bias, expedite management, and most importantly improve outcomes, the use of artificial intelligence (AI) has gained significant prominence in imaging analysis [1–4]. AI-driven methods have gained prominence in diagnosis (as exemplified by Computer-Aided Detection (CADe) systems and Computer-Aided Diagnosis (CADx) systems) for tuberculosis [5, 6], lung cancer [7, 8] and metastatic disease to the brain[9] and also been applied to multiple other areas of clinical need [4] including notably infectious diseases as described in the context of COVID-19 [10–12], internal medicine[13], diabetic retinopathy [14]. In oncology, AI methods are being explored in most cancer types including prominently in lung [8, 15], breast [16, 17], prostate [18], central nervous system cancers [13,19–26], and other malignancies [27]. There is ongoing progress in employing AI methods towards imaging for oncology treatment e.g., generating radiation therapy volumes [28], assessing treatment response [29], and understanding and communicating prognosis [19, 30]. Challenges relate to limited available data sets, variability in imaging changes over time present even within any one disease entity contingent on patient and disease-related factors, further augmented by a growing variability in analysis approaches [30–33]. In this descriptive review, we will review the imaging analysis workflow and examine how hand-crafted features also referred to as traditional Machine Learning (ML), Deep Learning (DL) approaches, and hybrid analyses that combine both approaches are being employed towards advancing AI-driven imaging analysis in central nervous system tumors.

### 1.1. The imaging analysis workflow

**Figure 1:**
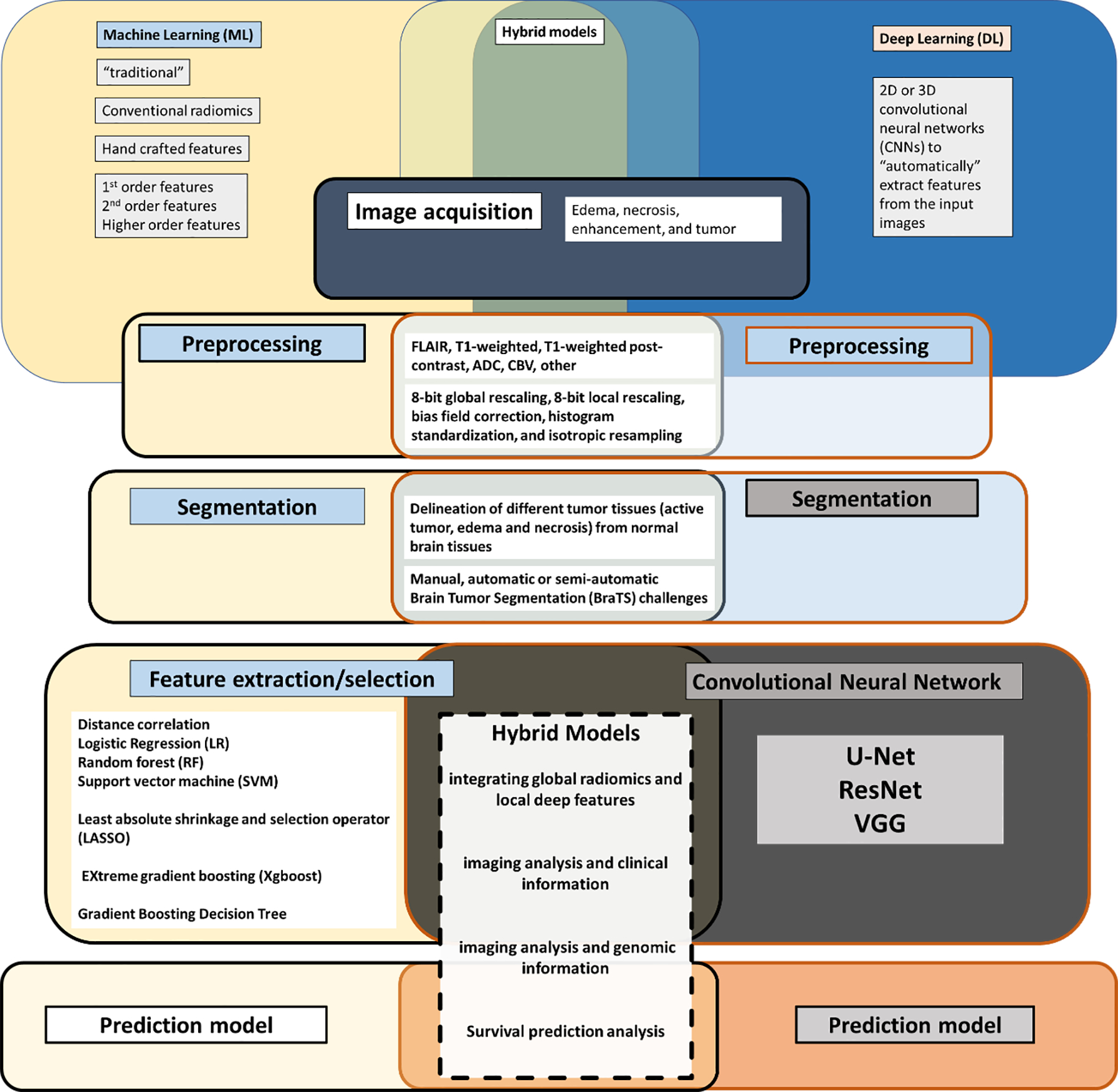
AI-driven image analysis workflow, traditional machine learning, deep learning and hybrid models

**Figure 2:**
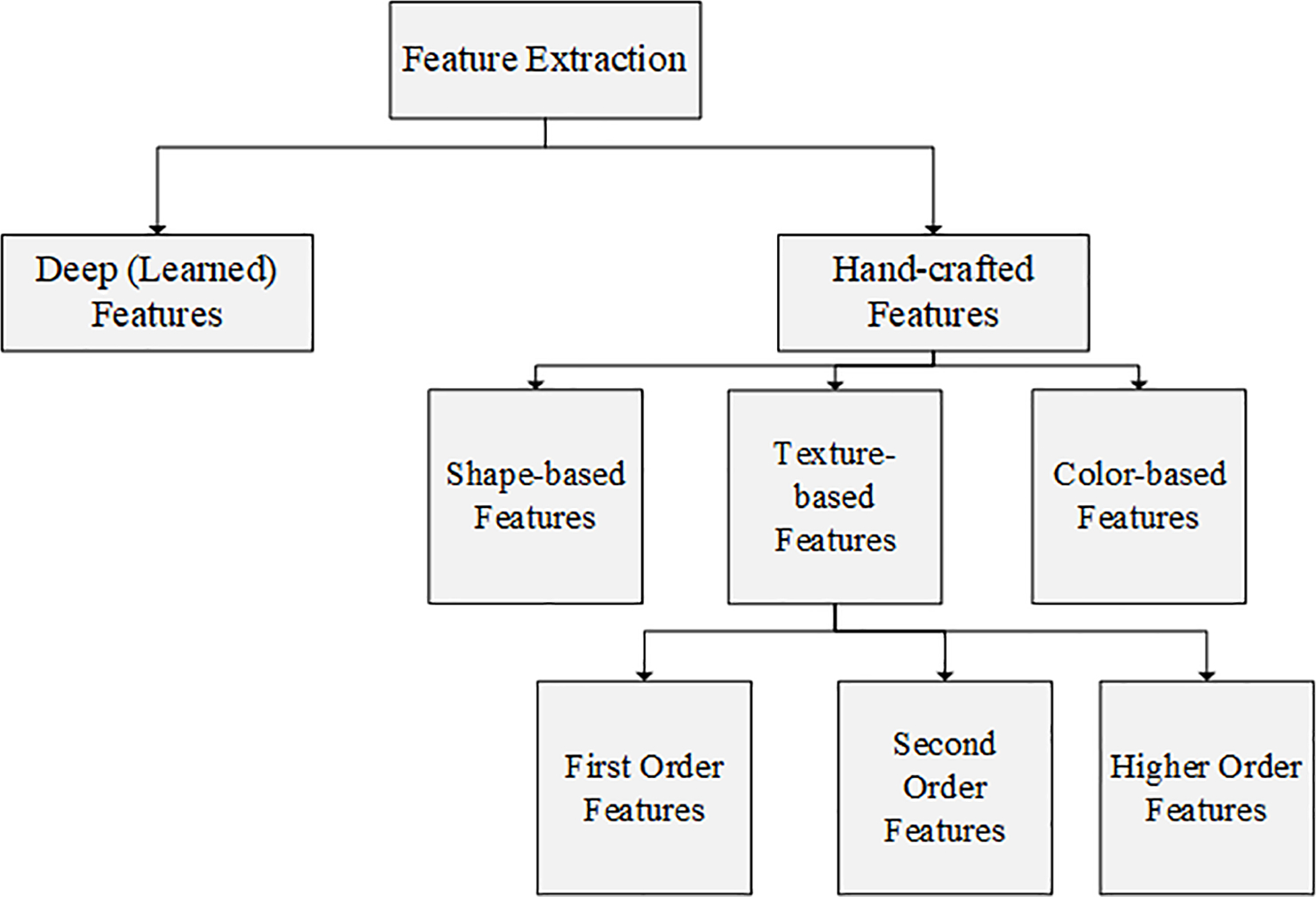
The overview of the feature extraction methods

To examine the variability in technical approaches to imaging analysis, a review and definition of the imaging analysis workflow are necessary (Figure 1). A number of publications have now reported on the workflow involved in harnessing quantitative data embedded in images for eventual analysis in a variety of clinical settings, in the context of the COVID-19 pandemic, and in multiple oncologic settings that are imaging driven [2, 11, 16, 25, 34, 35]. All aspects and terms involved in the image analysis workflow continue to evolve and grow in complexity (Figure 2). Each aspect of the workflow is the subject of extensive ongoing research and publications [3, 36–39]. In a recent review of publications related to DL and imaging analysis spanning 2012 to 2020, brain, prostate, and diabetic retinopathy-related studies were mature research topics in the field with breast- and lung-related studies in a stage of rapid development. Segmentation and classification tasks were the primary purposes of DL and imaging analysis papers [3]. The rapid evolution and growth of publications in the field, has made it more challenging for clinicians to stay connected to the complexity of AI-driven analysis and to transparently evaluate different approaches [2, 37, 40, 41]. This is particularly difficult since there is significant heterogeneity in the approach taken, with some clinical aspects analyzed using traditional Machine Learning (ML), Deep Learning (DL), or a combination of both. Various data sources are being employed, at times discrepant features identified by means of alternate methods, heterogeneous validation of results, and limited reproducibility persist.

## 2. Image Preprocessing

The imaging analysis workflow begins with image acquisition and registration followed by pre-processing to address noise, inhomogeneity, variability in intensity (e.g., different vendors), in central nervous system cancers imaged with MRI, capturing non-brain tissue (e.g., skull). Pre-processing techniques include therefore skull stripping, de-noising, bias field correction, and registration all of which are used to prepare brain MRI data for automatic brain tumor segmentation and analysis [42]. The importance of pre-processing as the cornerstone of all steps that follow it, cannot be understated [30, 33, 43–45]. With respect to the impact of preprocessing on the scanner dependence, several preprocessing techniques have been employed in the literature: 8-bit global rescaling, 8-bit local rescaling, bias field correction, histogram standardization, and isotropic resampling [45]. Um et al. employed two independent GBM cohorts (50 cases from the Cancer Genome Atlas Glioblastoma Multiforme (TCGA-GBM) dataset and 111 cases from their institution), each case consisting of 3 MRI sequences (FLAIR, T1-weighted, and T1-weighted post-contrast) and found that histogram standardization contributed the most in reducing radiomic feature variability reducing the covariate shift for three feature categories and successfully discriminating patients into groups of different survival risks [45]. The effect of image preprocessing with respect to intensity inhomogeneity correction and noise filtering and its impact on the robustness and reproducibility of radiomics features was examined by Moradmand et al. who extracted 1461 radiomics features from multimodal MR images of glioblastoma tumors focusing on GBM subregions (i.e., edema, necrosis, enhancement, and tumor) and using FLAIR, T1, T1C, and T2 volumes for five preprocessing combinations (in total 116 880 radiomic features) and found that necrosis regions had a higher number of highly robust features as compared to edema [44]. They concluded that high reproducible features were more readily identified after bias field correction and bias field correction followed by noise filtering as compared with noise smoothing or noise smoothing followed by bias correction [44]. By contrast, Baessler et al., investigated both reproducibility and repeatability of radiomic features using a physical phantom scanned using different sequences and found that radiomic features extracted from FLAIR (Fluid Attenuated Inversion Recovery) images were more repeatable than features from T1- and T2-weighted images [43]. These studies reinforce the importance of preprocessing in terms of the robustness and reproducibility of MRI-based radiomic features and the identification of generalizable and consistent preprocessing algorithms [33].

## 3. Segmentation

**Figure 3:**
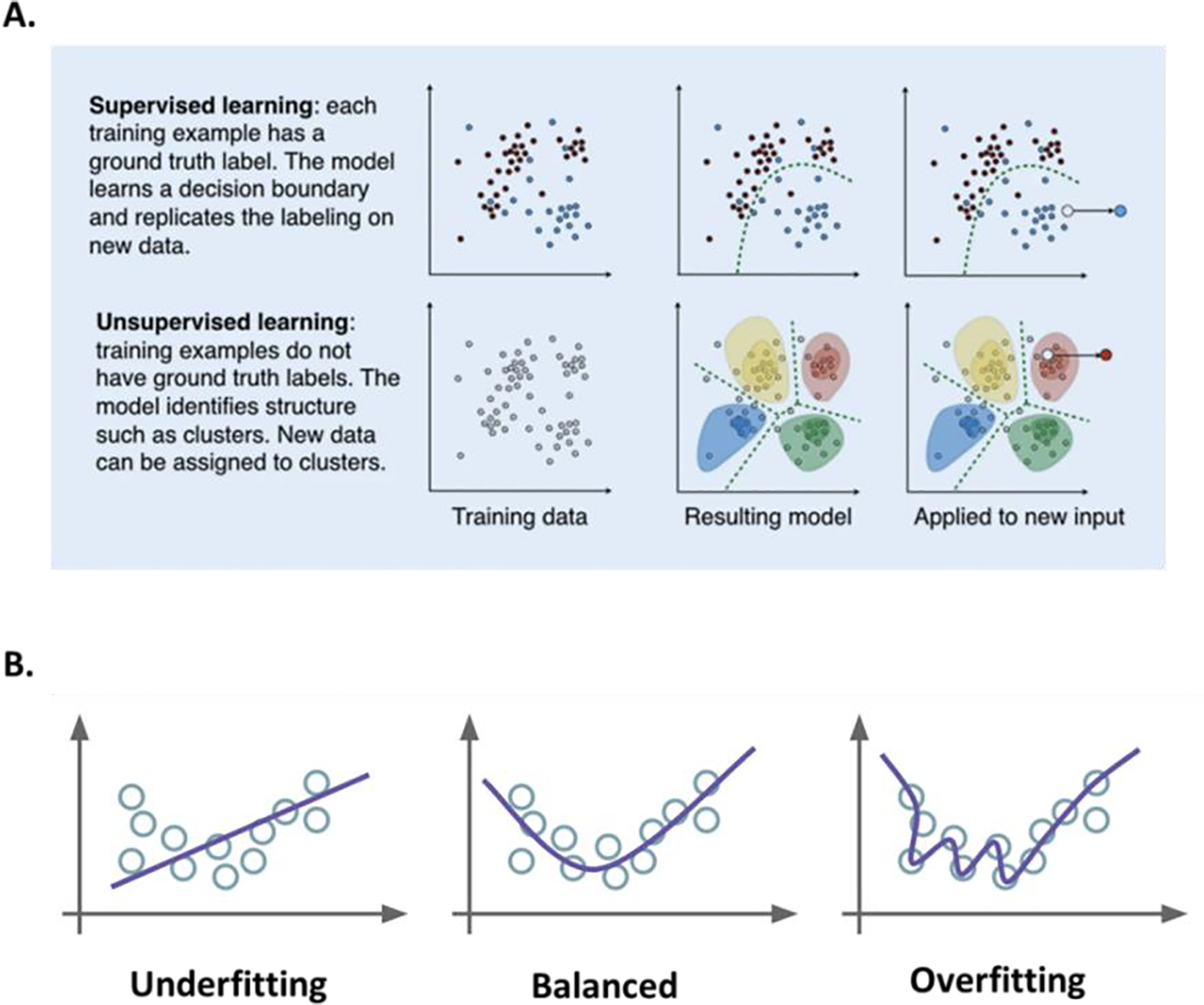
A) Supervised and unsupervised machine learning [55]. B) Data overfitting and underfitting [56].

Segmentation follows pre-processing and can be a manual or automated process depending on the human involvement, often increasingly achieved using deep learning [3, 20, 30, 42, 46–53]. Segmentation in and of itself is a significant area of active research and evolution [51]. As a crucial initial step, in neuro-oncology imaging analysis, it allows for the delineation of different tumor tissues (active tumor, edema, and necrosis) from normal brain tissues (Figure 1). Variability in approach here is significant as manual segmentation as might be performed by a clinician is time-consuming, requires significant expertise, and is subject to human interpretation. Automatic or semiautomatic methods are increasingly employed, and the field has been growing in part spurred on by the Brain Tumor Segmentation (BraTS) challenges [54]. Depending on whether the segmentation method initially uses annotated data to eventually automate the process using ML or DL, it may be supervised (using annotated data), unsupervised (no training or annotated data is employed) or a hybrid of both (Figure 3A) [55, 56]. Automated segmentation can involve classic radiomic machine learning methods such as comparing Support Vector Machine (SVM) or be performed using Deep Learning (DL) with Convolutional Neural Networks (CNN) (DL) and data has shown that both approaches may be reliable and fast, however, CNN technique may outperform SVM in the accuracy of segmentation with requirements of significantly enlarged data set, long computation time and high-performance computer [57]. Singh et al. produced a thorough review of publicly available automatic brain segmentation methodologies, machine learning models, recent advancements, and their comparison [31]. A discussion of the terms [58] including overfitting (i.e., model will only memorize the training data, suppressing the ability of CNN to generalize to unseen invariant data) and underfitting (i.e. model is not adequately trained and cannot capture the relationship between features and target labels) (Figure 3B) [56], is important to conceptualize the potential pitfalls embedded in AI approaches as related to any aspect of the imaging analysis workflow. Overfitting and underfitting represent potential modeling errors. Overfitting, as opposed to underfitting, is more often identified since it can be the result of small datasets that attempt to “stretch” the conclusion to previously unseen data. Overfitting can be addressed by employing large data sets (the size of which is under debate) and may also be addressed by employing data augmentation where the data sets may be artificially grown using image transformations [59]. Overfitting can also be the result of overtraining and this aspect can be addressed through cross-validation (data resampling method to assess the generalization ability of predictive models and to prevent overfitting)[60]. Underfitting on the other hand may be the product of inadequate training or insufficient features in the model. Both modeling errors can be identified by assessing the model`s performance metric [61].

### 3.1. Feature extraction, feature selection, and the creation of machine learning models

Feature extraction, feature selection, and the creation of machine learning models for predictive and prognostic applications follow segmentation and are most often the focus of discussion since the perception is that it is this step that is most closely connected to clinically meaningful results [30]. Feature extraction is a process frequently used in pattern recognition and image processing and is applied to reduce the size of the input data and the number of required resources and to obtain informative and discriminative data (Figure 1). Feature extraction methods can be split into hand-crafted or deep (learned) features concerning machine or deep learning approaches applied. Hand-crafted features can be shape-based, texture-based, and/or color-based (Figure 2). On the other hand, the feature selection stage provides a dimensionality reduction that can be defined as finding a subset of x features from all features set y, where x ≤ y. It aims to reduce the number of variables for the following step (i.e., machine learning). Feature selection methods are essentially grouped into three categories based on evaluation of objective function concerning information content, or predictive models: filters, wrappers, and embedded methods [62]. Methods for feature extraction and selection are actively evolving. In ML feature selection measures carry significant importance. Increasingly data is evolving showing that ML-based imaging analysis is vulnerable to feature selection [61, 63]. In a recent study where radiomics-based ML algorithms in Differentiating Glioblastoma (GBM) from primary central nervous system lymphoma (PCNSL) were evaluated, 5 selection methods (distance correlation, random forest, Least Absolute Shrinkage and Selection Operator (LASSO), eXtreme gradient boosting (Xgboost), and Gradient Boosting Decision Tree) and 3 radiomics-based ML classifiers Linear Discriminant Analysis (LDA), Support Vector Machine (SVM), and Logistic Regression (LR)) were compared [63]. The authors noted that the most optimal discriminative performance was observed among all classifiers when combined with the suitable selection method. For LDA-based models, the optimal one was Distance Correlation + LDA with AUC of 0.978. For SVM-based models, Distance Correlation + SVM was the one with highest AUC of 0.959, while for LR-based models, the highest AUC was 0.966 established with LASSO + LR [63]. In a literature review spanning 2013 to 2018, LR and LASSO were the two most used techniques for feature selection [64]. Following a recent metaanalysis of radiomic studies wherein the primary outcome was the degree of repeatability/reproducibility of a radiomic feature with the secondary outcomes being the impact of image acquisition and reconstruction settings, preprocessing steps, and tumor segmentation on the reliability/reproducibility of radiomic features and the metrics used for reporting on reliability/reproducibility, Pfaehler et al. proposed a radiomic reporting checklist to evaluate the quality of reporting of analyzed studies [33]. In terms of convolutional neural network-derived networks, U-Net, ResNet, and VGG are the most frequently used [3, 65, 66]. GAN-derived networks were widely developed and applied in 2020, and transfer learning was highlighted in the COVID-19 studies [3]. In-depth discussion of these techniques is beyond the scope of this manuscript but has been exhaustively published on and important references are noted with excellent recent reviews Sahiner, Greenspan, Shin [65, 66]. From a clinician standpoint, there is a significant evolving challenge in grasping rapid progress and complexity of the techniques employed as part of the image analysis workflow, and a growing body of papers have been aimed at expanding on the nuances required to evaluate publications and conclusions based on AI-driven data analysis [27, 41, 67]. According to a recent review, the annual growth rate in the number of published papers was 177.82% and radiomics was found to be at a more mature stage for lung, breast, and prostate cancers than for other sites [3, 64]. Radiomics studies primarily focused on radiological characterization and monitoring [64]. Of note, non-clinical researchers without a medical background dominated radiomics studies (70.52%), the vast majority of which only highlighted positive results (97.80%) while downplaying negative findings [64].

### 3.2. Traditional machine learning methods based on hand-crafted features

**Figure 4:**
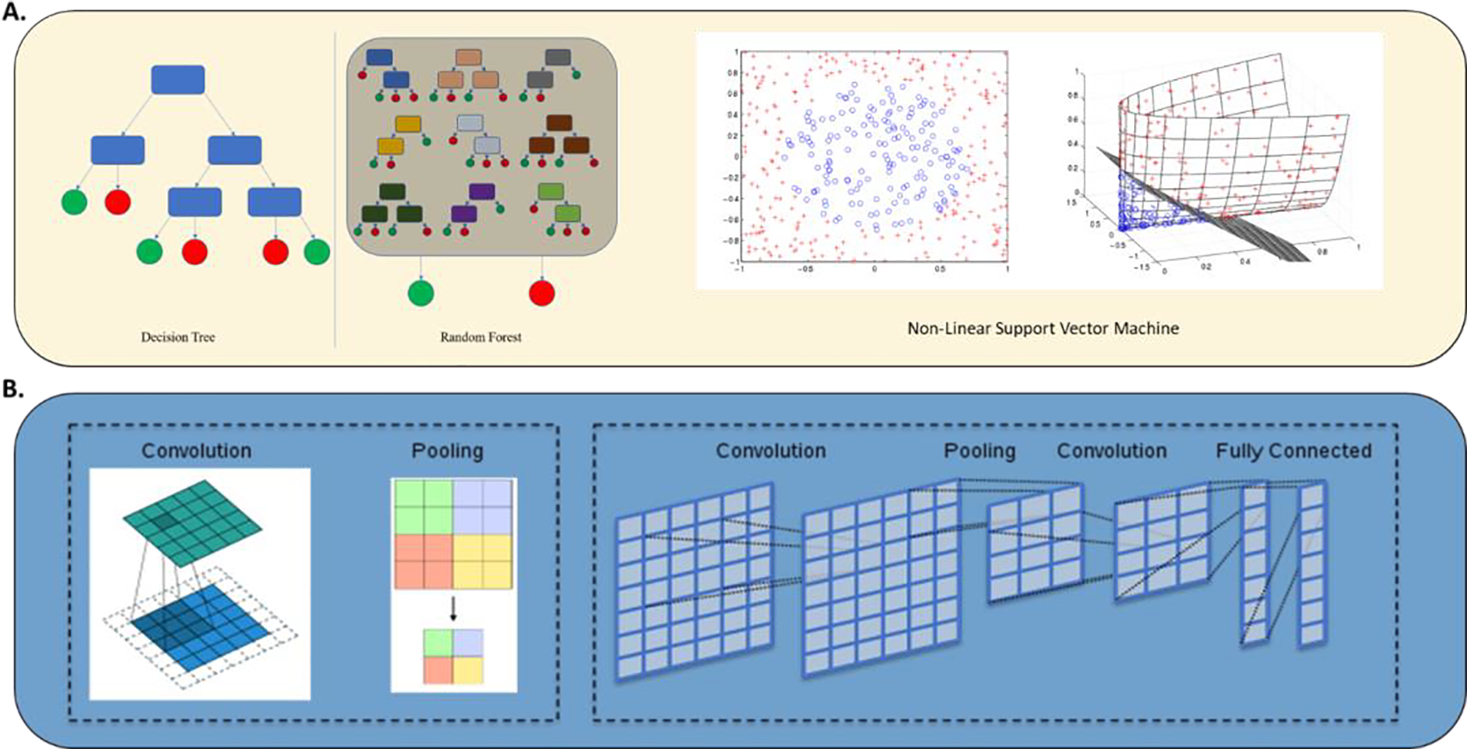
A) Traditional machine learning: Decision tree, Random Forest (RF) and Non-linear Support Vector (SVM) [69, 70]. B) Deep learning in medical image processing - Convolutional layers and pooling layers are typically combined in an alternating manner to construct convolutional neural networks (CNNs) [94, 95].

Radiomics is defined as the extraction of “hand-crafted” features from routine radiological scans (X-rays, CT, MRI, and PET) that quantitatively capture the textural and morphological characteristics of a given tumor [30]. This is also often referred to as “traditional” radiomic analysis. This type of extraction of information from imaging converts an image to a predefined list of attributes, such as shape, intensity, texture, is referred to as “feature extraction” in the context of traditional machine learning [68]. These “hand-crafted” features can then be used in traditional learning algorithms, such as Random Forest (RF), Support Vector Machines (SVM) and k-nearest neighbors (Figure 4A) [69, 70]. Using imaging as an input for traditional machine learning algorithms requires an additional feature extraction step, making it distinct from Deep Convolutional Neural Networks (DCNNs) which learn image features as an implicit step in the process of optimizing output performance accuracy, discussed in the next section [71, 72]. Hand-crafted features are obtained using software such as Pyradiomics which is an open-source python package for the extraction of radiomics features from medical imaging [73]. A recent meta-analysis revealed that Pyradiomics was used in eight human studies and one phantom study, and was the most frequently used software, with others using open-source software (CGITA, MaZda, LifeX, IBEX) and others using Matlab [33]. In imaging analysis of central nervous system tumors, analyses that employ traditional radiomic analysis have been applied to combine imaging and molecular markers notably O6-methylguanine-DNA methyltransferase (MGMT) [74–77], IDH [74, 78–80], 1p19q [81, 82], H3K27M [83]. The diagnostic performance of radiomics using ML algorithms to predict MGMT status in glioma patients was the subject of a comprehensive literature search of PubMed, EMBASE, and Web of Science until 27 July 2021 [75] which identified 15 studies with 1663 patients and documented a pooled sensitivity and specificity of ML for predicting MGMT promoter methylation in gliomas of 85% and 84% in the training cohort (n=15) and 84% and 78% in the validation cohort (n=5) with an AUC of 0.91 in the training cohort and 0.88 in the validation cohort concluding that ML can predict MGMT promoter methylation status in glioma with a higher performance than non-machine learning methods [75]. Diffusion- and perfusion-weighted MRI radiomics models that may predict Isocitrate Dehydrogenase (IDH) mutation and tumor aggressiveness in diffuse lower grade glioma were explored by Kim et al. who compared multiparametric and conventional MRI radiomics models using the area under the receiver operating Characteristics Curve (AUC) while optimizing the multiparametric MRI radiomics model using a random forest feature selector, finding that for IDH mutation, multiparametric MR radiomics showed similar performance (AUC 0.795) to the conventional radiomics model (AUC 0.729) but in tumor grading, the multiparametric model with Attenuated Diffusion Coefficient (ADC) features showed higher performance (AUC 0.932) than the conventional model (AUC 0.555). This was confirmed following independent validation with AUCs of 0.747 for IDH prediction and 0.819 for tumor grading with the multiparametric MRI radiomics model [84]. Non-invasive genotype prediction of chromosome 1p/19q co-deletion in lower-grade gliomas [81] was examined in a retrospective study (277 patients histopathologically diagnosed with Lower-Grade Glioma (LGG)) that included clinical parameters and employed radiomics analysis by extracting 647 MRI-based features using random forest algorithm to generate a radiomics signature for predicting 1p/19q co-deletion in the training cohort (n = 184). A combined model, incorporating both the radiomics signature and related clinical factors, was also generated. The radiomics model was highly effective with AUCs of 0.887 and 0.760 for training and testing respectively, and it outperformed the clinical model with equally excellent results obtained for the combined model (AUCs 0.885 and 0.753 on training and validation cohorts respectively)[81]. In this study, clinical factors did not provide additional improvement for the prediction. Another study by Kha et al. examined a model based on data extracted from The Cancer Imaging Archive (TCIA), including 159 LGG patients with 1p/19q co-deletion mutation status and XGBoost as the baseline algorithm combined with SHapley Additive exPlanations (SHAP) analysis and selected the seven most optimal radiomics features to build the final predictive model which achieved accuracy of 87% and 82.8% on the training set and external test set, respectively [82]. Scenarios of highly curated data sets of more homogenous molecular and histological classification as exemplified by H3K27M mutation analysis in pediatric high-grade gliomas, radiomics applications in meningioma, and in pituitary neuroendocrine and sellar tumors are increasingly reported [24, 83, 85–90]. Wu et al. employed MRI radiomics and clinical features to preoperatively predict H3K27M mutation in pediatric high-grade gliomas using 9 radiomics features to construct the radiomics signature and showed a favorable discriminatory ability in training and test sets with an AUC of 0.95 and 0.92, respectively [83]. Ring enhancement was identified as an independent clinical predictor and the model had excellent calibration and discrimination in training and testing sets (AUC 0.95 and 0.90 respectively). Radiomic approaches have been combined with other data sets eg. Histopathology [91] to achieve superior results. Rathore et al. employed high and low-grade tumors from The Cancer Imaging Archive (original images acquired 1983–2008) to extract an extensive set of engineered features (intensity, histogram, and texture) from delineated tumor regions on MRI and histopathologic images and used Cox proportional hazard regression and SVM models to MRI features only, histopathologic features only and combined MRI and histopathologic features and found that the combined model had higher accuracy in predicting OS as compared to either model in isolation (AUC 0.86) [91]. Ultimately, traditional ML-based methods do depend on several aspects including segmentation which does introduce both a component of workload as well as bias since the segmentation itself and the methods involved do dictate the signal that is eventually measured and interpreted [19, 30, 38, 48]. It should also be noted that in the context of central nervous system tumors and other cancers treated with radiation therapy, the tumor volumes themselves are manually delineated to allow for targeting of the tumor with radiation therapy. A connection between segmentation and oncologic management that has led to a number of avenues exploring auto-segmentation to improve reproducibility and increase efficiency in the clinic [23, 92, 93]. Hand crafted methods and traditional radiomic continue to coexist with DL, discussed next however they both suffer from several limitations and are undergoing active evolution [33].

### 3.3. Deep learning-based methods

In deep learning, image features can be “learned” implicitly through the iterative process of optimizing prediction performance/accuracy (Figure 4B) [94, 95]. While DL approaches take images as input without the need to reduce them to a predefined, expert-curated list of attributes, hence potentially mitigating human bias, they can suffer from difficulties with respect to clinical applicability of conclusions and require large data sets. The data sets that are employed may themselves represent a source of bias since they are originating from a few select institutions. The bias inherent in these sets relates to image acquisition but also to the patient and clinical features that may be embedded in the selection of the data set itself. DL has been employed in segmentation tasks [46, 52], organ and lesion detection [4], lesion, tissue and tumor classification tasks [4, 53, 96], diagnosis [4], prognosis, staging and outcome prediction [4, 30, 46, 71, 78, 97, 98], image registration and quality assurance [4]. Segmentation is arguably the task that has been explored the most [34]. CNN typically employs three major techniques to medical image classification: training the CNN from scratch, using off-the-shelf pre-trained CNN features, and conducting unsupervised CNN pre-training with supervised finetuning [71]. A classification task of significant importance in central nervous system tumors is molecular subtyping such as in the context of diffuse glioma. Li et al employed preoperative multiparametric MRI in 1016 diffuse glioma patients randomly divided into the training (n = 780) and validation (n = 236) and generated predictive models based on radiomics and DCNN finding that while both the radiomics and DCNN models could preoperatively predict the molecular subtypes of diffuse gliomas, the DL model performed better in most circumstances with AUCs of the DCNN models (0.85–0.89) [99]. The authors however also noted that the correlation between the radiomics features and DCNN features was low. The interplay between imaging and molecular characterization has also been explored using DL. Yogananda et al. employed brain MR imaging and corresponding genomic information from 247 subjects from The Cancer Imaging Archive and The Cancer Genome Atlas of which 163 had a methylated MGMT promoter and developed a T2WI-only network (MGMT-net) to determine MGMT promoter methylation status and simultaneous single-label tumor segmentation [77]. The network was trained using 3D-dense-UNets and demonstrated high classification accuracy in predicting MGMT promoter methylation status using only T2WI with the predictive ability for predicting MGMT methylation status with a sensitivity and specificity of 96.31% and 91.66% respectively and AUC of 0.93 [77]. In a separate study, a similar analysis was carried out for IDH mutation status reporting a sensitivity of 0.97, specificity of 0.98 and an AUC of 0.98[100]. DL-based methods are growing in scope and importance in imaging analysis for central nervous system tumors in particular with respect to diagnosis and classification reflecting the complexity and evolving understanding of molecular characterization of central nervous system tumors and the inability to label large-scale molecular data by human experts [19, 22, 25].

### 3.4. Hybrid approaches

In an effort to optimize the results of AI-driven analyses, hybrid models have increasingly been developed to harness the advantages of both ML and DL approaches [88]. Hybrid approaches can involve all or a combination of the steps, where each step involves a distinct ML/DL training and validation process, in the imaging analysis workflow (Figure 1). Preprocessing can involve both ML and DL methods and segmentation has for some time been approached with hybrid methods that can start out with manual and/or and ML and then progress to auto segmentation, the product of which is then employed towards further data analysis and clinical conclusions. Fu et al. used 4 pre-operative MRI images of 285 patients with glioma (210 GBM, 75 LGG) and manually drawn tumor contours to train and validate a 3-dimensional CNN and then applied the trained CNN to the remaining 163 patients with GBM to generate their auto segmented tumor contours[34]. Both handcrafted and DL-based radiomic features were extracted from the autosegmented contours using explicitly designed algorithms and the pre-trained CNN respectively and cox regression models were trained to construct the handcrafted and DL-based signatures. The CNN achieved a mean Dice coefficient of 0.86 as compared to the handcrafted signature (0.64) and the DL-based (0.67) however the DL-based signature successfully stratified testing patients into 2 prognostically distinct groups [34]. Ning et al. also analyzed the feasibility of integrating global radiomics and local deep features based on MRI to create a grading model [101]. 567 patients who had undergone postcontrast enhanced T1-weighted and T2 FLAIR MRI (211 patients with glioblastomas (GBMs) and 356 patients with low-grade gliomas (LGGs)) were included and radiomics and deep features were extracted. The model based on the combination of radiomics and deep features outperformed the models based only on either radiomics or deep features (AUC 0.94 and 0.88 for the validation and the independent testing cohort respectively) [101]. It is also increasingly becoming clear that CNN may extract drastically different features than radiomics models [102]. Limitations that are recognized include the selection of tumor imaging volumes selected for CNN (eg. 2.5D input format with skipped slices, vs 3D tumor volume for radiomics input) [99,102]potentially resulting in biased feature comparisons and therefore biased feature correlations between radiomics and DL.

A special category is a hybrid approach of combining ML and DL in imaging analysis with clinical information. Results in this space have been mixed. Guo et al. aimed to explore whether multiparametric MRI-based radiomics combined with selected blood inflammatory markers could effectively predict the grade and proliferation in glioma patients and found that the radiomics signature demonstrated good performance in both the training and validation cohorts, with AUCs of 0.92, 0.91, and 0.94 and 0.94, 0.75, and 0.82 for differentiating between low and high-grade gliomas, grade III and grade IV gliomas, and low Ki-67 and high Ki-67, respectively, all better than the clinical model [103]. The AUCs of the combined model were 0.93, 0.91, and 0.95 and 0.94, 0.76, and 0.80, respectively, and ultimately both the radiomics signature and combined model outperformed the clinical model. Interestingly, although the clinical factors did not improve the prediction of the grade and proliferation index, the stability of the model was improved. By comparison, Wang et al. used a training cohort of 168 HGG patients and a validation cohort of 42 HGG patients extracting 1284 radiomics features and 8192 deep features (extracted via transfer learning) using Least Absolute Shrinkage and Selection Operator (LASSO) regression to select features, and integrating this with clinical predictors, found that the radiomics and deep signatures were significantly associated with HGG patients’ survival time and the signature derived from the synthesized radiomics and deep features showed a better prognostic performance than either the radiomics or deep features alone [104].

These studies raise the question of the variability of clinical data collection and the extent and type of clinical information being included that is necessary to improve model pred-iction beyond image-based analysis and the importance of feature extraction methods. With respect to survival prediction and potential hybrid methods that combine it with image analysis, DeepSurv, a DL based-survival prediction algorithm, has already been successfully employed in oncologic settings (oral cancer [105], renal cell [106]) and may be combined with ML and DL approaches that harness imaging information in central nervous system tumors. Survival prediction is an active area of research that will require multiple data inputs to be successful and imaging analysis will be a paramount source of data in this area [39].

## 4. Conclusions

Significant variability persists in AI-driven imaging analysis at all levels of the workflow all of which are subject of ongoing research resulting in a significant number of publications. ML, DL, and hybrid approaches coexist, and their combination may produce superior results although data in this space is as yet novel, and conclusions and pitfalls have yet to be fully explored. There is a growing technical aspect that may become increasingly separated from the clinic and hence clinician involvement is much needed to guide progress and ensure that conclusions derived from AI-driven imaging analysis approaches reflect that same level of scrutiny lent to other avenues of clinical research.
